# The relative role of executive control and personality traits in grit

**DOI:** 10.1371/journal.pone.0269448

**Published:** 2022-06-22

**Authors:** Nuria V. Aguerre, Carlos J. Gómez-Ariza, M. Teresa Bajo

**Affiliations:** 1 Department of Experimental Psychology–Mind, Brain, and Behavior Research Center (CIMCYC), University of Granada, Granada, Spain; 2 Department of Psychology, University of Jaen, Jaen, Spain; University of Zurich, SWITZERLAND

## Abstract

Although grit is predictive of wellbeing, educational achievement, and success in life, it has been conceptualized as largely distinct from cognitive ability. The present study investigated the link between grit and executive functions since regulation abilities might underlie the expression of grit. A hundred thirty-four people were administered personality questionnaires (grit, impulsiveness, and mindfulness) and four experimental tasks tapping into Miyake’s and Braver’s models of executive functioning (including measures of flexibility, inhibition, working memory, and control mode dimensions). Multivariate analyses showed that two composite scores (trait and executive functioning) were reliably predictive of grit, although it was the trait composite (characterized by low impulsivity and high mindfulness) that explained more variance. Importantly, gritty participants did not demonstrate enhanced executive functioning. Instead, they exhibited a different pattern of performance that might be reflecting a cautious profile of control, characterized by paying attention to all available information, less reliance on previous contextual cues but sensitive to conflicting information of the current context. These findings converge with Duckworth’s idea that high grit people do not necessarily have a greater cognitive capacity. Rather, they use it in a different way.

## Introduction

To crown the top of Mt. Everest is not an easy challenge, nor is it to finish a PhD. Some people will spend a good few years of their lives training and working day after day to achieve these challenges, whereas other people prefer short-term goals. To engage and achieve long-term goals require grit, a personality trait that refers to the tenacious pursuit of a dominant superordinate goal despite setbacks [1–3]. In the last decade, grit has received attention within the discipline of positive psychology as well as in the fields of motivation and education. Research within these disciplines has indicated that grit significantly contributes to wellbeing [4], (i.e. lower depression [5, 6], reduction of risk of suicidal ideation [7, 8]) and predicts success in work and personal life [9, 10], as well as academic achievement [3, 11].

As mentioned, grit is conceptualized as a personality trait reflecting perseverance and passion for long-term goals and it is usually assessed with the Grit Scale [12], which comprises two factors: perseverance of effort and consistency of interest. Perseverance of effort reflects effort toward one’s enduring or superordinate goal (i.e., ‘Setbacks don’t discourage me’), whereas consistency of interest refers to the ability to focus on a small set of relevant goals related to the pursuit of a larger, more important objective (i.e., ‘New ideas and projects sometimes distract me from previous ones’, inversed item). A number of studies have examined the relationship between grit and other personality factors. For example, some studies have shown a positive relationship between grit and conscientiousness (tendency to be hardworking and self-disciplined), self-regulation (tendency to appraise before and during performance), and engagement (tendency to get deeply involved in an activity) [13, 14]. Similarly, grit seems to be negatively related to impulsiveness, which refers to the tendency to perform swift actions without conscious judgment [15, 16], and positively related to mindfulness [17–20]. Mindfulness is usually defined as a state of non-judgmental attention toward the present experience [21] and thought to comprise five factors as measured with the FFMQ (Five Facets Mindfulness Questionnaire) [22]; namely, observing, describing, acting with awareness, non-judging of inner experience and non-reactivity to inner experience. Importantly, when engaged in a long-term goal, temptations and distractions may often appear that involve momentary salient cues of immediate gratification. In these cases, the conscious attention towards the present moment that is characteristic of mindfulness may make people more aware of the presence of conflicting goals and prevent them from automatically choosing a more hedonically pleasant option. In fact, previous work has demonstrated that mindfulness predicts the development of grit [18], and some differences have been observed between non-western and western cultures [19]. In the latter, acting with awareness and non-judging facets were found to predict grit more strongly [18]. A secondary goal in the present study was to replicate the already observed associations between grit, impulsiveness, and mindfulness in a Spanish sample of diverse backgrounds. The main goal was to gain understanding of the relative role of trait variables and cognitive (executive) functions in the description of grit.

While predictive of academic success, grit has been conceptualized as largely distinct from cognitive ability [3, 23, 24]. Indeed, grit and cognitive ability have been shown to be orthogonal in several studies [3, 9]. Nevertheless, it should be noted that these studies have mainly employed admission tests to measure cognitive functions [24] and very few studies have adopted an experimental approach to look into the cognitive bases of grit [25, 26]. Kalia et al. [25] used the Attention Network Test (ANT) to examine the relationship between grit and different attentional functions (alerting, orienting, and executive attention). Their results only showed an association with the alerting function, so that the perseverance of effort facet of grit negatively correlated with the alerting effect (the benefit in reaction time that usually follows the presentation of an alerting cue as compared with no alerting cue), which they attributed to either better sustained attention or less sensitivity to warning cues by gritty individuals. More recently, Kalia et al. [27] found a positive association between the perseverance of effort facet of grit and performance on difficult Sudoku problems that was mediated by reduced cognitive flexibility (as measured by the Wisconsin Card Sort Test). Thus, those participants who scored higher in grit (and exhibited less cognitive flexibility) persisted more and performed better when solving hard problems. Although these results suggest that certain profiles of cognitive functioning could characterize gritty people, they do not specify the cognitive mechanisms underlying grit. The present study aimed to explore whether grit is related to executive functioning by using a wide range of experimental tasks based on two well-established frameworks of executive functioning.

Executive functions (EF) refer to a variety of control mechanisms in charge of guiding behaviour in a goal-driven manner to deal efficiently with changing environments [28], and, in principle, it seems reasonable to think that these control functions may underlie the development and expression of grit. On the one hand, personality traits that strongly relate to grit have been shown to be associated with executive functions (self-regulation [29]; impulsiveness [30]; mindfulness [31, 32]). On the other hand, activity in specific regions within the prefrontal cortex (PFC), which has been systematically shown to underpin executive functioning [33], has been related to grit [34–37]. However, no previous study to date has directly examined the relationship between grit and executive control. In an attempt to fill this gap, the main aim of the present study was to explore the potential link between grit and executive functions.

A classical classification of EF is the one proposed by Miyake et al. [28, 38], who identified three different executive control functions that conformed an unique construct while being clearly separable from each other: switching, inhibition, and working memory updating. Switching involves shifting flexibly between tasks or mental sets; inhibition entails the capacity to resist interference or for conflict resolution; and updating refers to the monitoring of information in working memory together with its revision to replace old items that are no longer relevant with newer relevant ones. These three executive components are usually measured with task-switching, conflict, and working memory tasks. These tasks provide indexes for 1) switching cost (SC), which represents worse performance in trials involving switching between different tasks as compared with trials with task repetitions [39, 40]; 2) conflict cost (CC), an inverse estimate of inhibitory control, whereby responses to incongruent trials are less accurate and slower than responses to congruent trials [41], and 3) working memory (WM) index, measured as a result of the number of items successfully stored in memory while accurately performing a mental operation task [42].

While the framework proposed by Miyake et al. [28, 38] has been very useful in classifying functions and tasks as well as in studying individual differences when confronting everyday challenges [43], other theoretical frameworks emphasizing more dynamic manners of facing these challenges have also been helpful. For example, the dual mechanisms of cognitive control (DMC) account proposed by Braver [44, 45] posits that two different cognitive control modes may be put into work: 1) a proactive mode, which acts by actively maintaining task-relevant information in a sustained manner to direct behaviour in accordance with internal goals; and 2) a reactive mode, which relates to the detection and resolution of interference at the time it occurs. The interaction between these two control modes is dynamic so that people might be more prone to one or another mode [46] and some situations might favour one mode over another [47, 48]. Also, it is the dynamic coordination of the two modes of control that allows individuals to be more adaptive in changing situations [49].

A well-established task to estimate the individuals’ control mode preference is the AX-Continuous Performance Task (AX-CPT) [44], wherein participants are instructed to respond to a probe on the basis of a contextual cue that signals a specific response in 70% of the trials (AX), while in the remaining trials (AY, BX and BY) a ‘no’ response is required either by cue or probe indication. A proactive control tendency is signalled by more errors when the contextual cue to respond ‘yes’ appears but a non-target probe forces an alternative response (AY). In contrast, reactive control is characterized by increased errors when the contextual cue signals a ‘no’ response but the probe elicits the dominant response (BX). The participants’ proactive vs. reactive tendency is calculated by the Behavioural Shift Index (BSI) wherein higher scores reflect a proactive tendency [44, 50]. In the present study, we used classical EF tasks following the Miyake et al.’s classification [28] as well as the AX-CPT to examine the relationship between executive control components and grit.

In summary, with the present study we aimed to gain understanding of the relationship between executive functioning and grit but also considering personality traits. Specifically, we selected two personality traits (impulsiveness and mindfulness) and four executive control indexes (switching and conflict costs, working memory, and Behavioural Shift Index) to examine their relationship to grit. On the one hand, we expected to extend previous findings showing a negative association between impulsiveness and grit and a positive relationship between mindfulness and grit in a Spanish sample of healthy young adults [15, 18]. On the other hand, we wanted to shed light on whether 1) grit is orthogonal to cognitive ability, such as Duckworth proposes [3]; 2) gritty participants have greater cognitive capacity so that they perform better than low grit people, or simply 3) gritty people exhibit a different pattern of cognitive control than their low grit counterparts. Although our study is to some extent exploratory given the lack of previous research, we expected individuals scoring high on grit to exhibit better executive functioning. From the conceptualization of grit that was described above, it seems reasonable to expect that enhanced ability for conflict resolution (better inhibition), working memory capacity and proactive control would be linked to higher scores in grit since the efficient use of these processes would contribute to downregulating irrelevant goals while keeping in mind the selected superordinate objective. Also, enhanced ability for task switching would seem to be necessary to take alternative pathways towards the superordinate goal after a failure, even though the above-mentioned results from a recent study suggest that tenacity might work at the expense of cognitive flexibility [27]. To test these hypotheses, we first examined simple relationships between executive indexes, trait variables, and grit. Then, we performed an exploratory factor analysis that extracted two factors comprising trait and executive variables separately. Finally, we tested regression models from composite scores driven by the factors previously extracted over grit.

## Methods

### Participants

A hundred thirty-four people (*M*_*age*_ = 22.92, *SD*_*age*_ = 4.1, Range_Age_ = 18–33, 72% female) participated in the study in exchange for course credits (1/40min) or monetary reward (7€/1h). This sample has been previously reported in two related studies: One aimed to examine the relationship between mindfulness and cognitive control modes [51], and another one focusing on the electrophysiological correlates of grit [52]. All studies are part of a larger research project on individual differences so that participants were administrated several different experimental tasks and questionnaires along with two assessment sessions. Importantly, the studies focus on different issues without significant overlap concerning aims or findings.

We ensured we had sufficient sample size prior to running the experiment by performing a power analysis [53] on the basis of the correlation between grit perseverance of effort and alerting effect in Kalia’s second study [25]. Results showed that eighty-one participants would be enough to capture a reliable association with 90% power and alpha set at 5%. All participants were provided with general information about the study and gave written informed consent following the Declaration of Helsinki [54] prior to being administered any of the tasks. Approval was obtained from the Ethics Committee of the University of Granada (number 84/CEIH72015).

### Materials and procedure

Participants were assessed across two sessions of 90 and 120 min, respectively. All participants were tested individually in isolated cubicles. In the first session, four questionnaires and four experimental tasks were administered. The questionnaires were a translated version of the Grit Scale [12], the Spanish version of the Barratt Impulsiveness Scale (BISS-11) [55], the Five Facets Mindfulness Questionnaire (FFMQ) [56] and the Mindful Attention Awareness Scale (MAAS) [57]. The experimental tasks were the Cued Task-Switching Paradigm [39], a Stroop-like Conflict Task [41, 58], the Operation Span (O-Span) [42, 59] and a distractor version of the AX-CPT [44, 60]. During the second session, the participants were administrated a Stop-Signal Task [61] and a Selective Retrieval-Practice task [62] in addition to MRI and EEG recordings. The results of the second assessment session are intended to be included in a different forthcoming paper and, consequently, are not presented here. The present study only concerns the first assessment session. All questionnaires and experimental tasks were counterbalanced across participants. Stimuli presentation and data acquisition were controlled by E-prime experimental software [63].

#### Personality questionnaires

*GRIT*. The Short Grit Scale is an 8-item self-reported questionnaire that assesses two factors of grittiness: perseverance of effort (i.e., ‘I finish whatever I begin’) and consistency of interest (i.e., ‘My interests change from year to year’, inversed item). Answers to the items go from 1 (very much like me) to 5 (not like me at all). The highest score on this scale is 5 (extremely gritty) and the lowest score is 1 (not at all gritty). Cronbach’s α of the factors in the original scale goes from 0.60 to 0.79 [12] and they are 0.59 for perseverance of effort and 0.81 for consistency of interest in our sample. Importantly, a confirmatory factor analysis validated the factor structure of grit in the current sample (see Supplementary Material 1 in S1 File).

*BISS-11*. This is a questionnaire of 30-items composed of three scales of impulsiveness: cognitive impulsiveness (i.e., ‘I am a happy-go-lucky’), motor impulsiveness (i.e., ‘I do things without thinking’), and non-planned impulsiveness (i.e., ‘I plan tasks carefully’, inversed item). Items in BISS-11 are ranged from 1 (rarely/never) to 4 (almost always/always), with higher scores reflecting greater impulsiveness. The Cronbach’s α of this questionnaire is 0.83 [55] and it was 0.79 in our study.

*FFMQ*. This is a self-reported questionnaire that measures five facets of mindfulness in 39 items. The facets are: observing (i.e., ‘I notice the smells and aromas of things’), describing (i.e., ‘It’s hard for me to find the words to describe what I’m thinking’, inversed item), acting with awareness (i.e., ‘I snack without being aware that I’m eating’, inversed item), non-judging of inner experience (i.e., ‘I disapprove of myself when I have irrational ideas’, inversed item) and non-reactivity to inner experience (i.e., ‘I watch my feelings without getting lost in them’). Answers in the FFMQ go from 1 (never or very rarely true) to 5 (very often or always true) so that higher scores reflect higher degree of mindfulness. The reliability indexes of the factors go from 0.80 to 0.91[22, 56] and in the current sample the range was 0.78–0.90.

#### Experimental tasks

*Cued task-switching*. We employed an adaptation for adults of the cued task-switching paradigm introduced by Chevalier et al. [39] as a measure of switching. The task goal was to sort objects either by their shape or by their colour as indicated by a trial cue. In every trial, three screens were presented. In the first one, a fixation cross appeared in the centre of the screen with a jittered inter-trial interval between 1000–1200 ms. In the second screen a box surrounded by the task cue or a neutral cue was presented for 500 ms. In the third screen the two-dimensional target (8 x 6 cm; i.e., a blue balloon), surrounded by the task cue or a neutral cue, was presented in the centre of the screen until a response was entered or for up to 1200 ms. The neutral cue consisted of 12 beige squares that were not informative of the dimension of the target to respond to, whereas the task cue could be 12 beige different geometrical shapes that indicated a shape response, or 12 squares of different colours that indicated a colour response. The task was divided into three blocks with a different combination of shape and colour (balloon-airplane-blue-red; apple-banana-green-yellow; ball-bat-orange-purple). Critically, the timing of cue presentation was blocked and the order of the blocks was counterbalanced across participants. In the ‘proactive impossible’ block, the neutral cue was presented with the box and the task cue was presented with the target so that reactive control was required to give a correct answer. In the ‘proactive possible’ block the task cue was presented along with the box and remained visible after target onset so that either proactive or reactive control could be used. Finally, in the ‘proactive encouraged’ block there was an early presentation of the task cue (along with the box) and the neutral cue was presented along with the target so that proactive control was necessary to respond. Participants were required to give a response by pressing different keys with one of their four fingers (index and middle fingers of each hand), previously associated with each colour and shape. The task had 216 trials, 8 practice trials wherein feedback was provided and 64 experimental trials per block with pauses every 16 trials. In each experimental block, 32 trials were no-switch, the task was the same as the previous trial, and 32 trials were switch, the relevant task changed from the previous trial. Switch and no-switch trials alternated unpredictably. A switching cost index can be calculated by subtracting switch trials to no-switch trials on accuracy or the other way on RTs; the lower the SC the greater the flexibility of the participant [39, 40]. We used a SC based on accuracy in the proactive possible block because this is the neutral block and previous findings have shown it to be the most sensitive [51]. However, results regarding all blocks of the task are available in Supplementary Material 2 in S1 File. A split half analysis to test the reliability of the SC index in the current sample indicated a Spearman-Brown coefficient of 0.37.

*Conflict task*. This task was used to measure the capacity for conflict resolution (inhibition). The task employed was an adaptation of the original paradigm [41, 58] that requires participants to respond either to the direction of an arrow (pointing to left or right) or to the meaning of a word (‘left’ or ‘right’) that was placed inside the arrow. The answers were given by pressing either the ‘1’ key of the keyboard to respond ‘left’ or the ‘5’ key to respond ‘right’. A cue presented previously signalled if participants had to respond to the direction of the arrow or to the meaning of the word; the cue was either an ‘f’ (from ‘flecha’, arrow in Spanish) or a ‘p’ (from ‘palabra’, word in Spanish), respectively. Each trial consisted of the presentation of three consecutive items. First, a fixation point (a centred cross) was presented for 400ms. Then, the cue (‘f’ or ‘p’) appeared in the centre of the screen for 500ms. After this, the arrow containing a word was presented in the centre of the screen until a response was entered or up to 1500ms. The task had 8 practice trials in which feedback was provided plus an experimental block of 40 trials. Twenty trials were congruent (the direction of the arrow matched the meaning of the word: 10 left, 10 right), and the remaining 20 trials were incongruent (the direction of the arrow mismatched the meaning of the word: 10 left, 10 right). Half of the trials required a response to the word, while the other half required a response to the arrow. Different types of trials were presented randomly. An interference index (reflecting conflict cost) was computed by subtracting reaction times on incongruent trials from reaction times on congruent trials [58]. The split half reliability for the conflict cost in the present sample was 0.72.

*O-SPAN*. This task was used to assess working memory capacity. We employed the Spanish version of the original task introduced by Turner and Engle [42, see also 58]. This is a dual working memory task wherein participants had to verify mathematical equations while keeping in memory sets of words of increasing set sizes. Stimuli were presented centrally on a white background. In each trial, an operation-word pair was presented. First, a solved mathematical operation [i.e., (14/2) + 2 = 9] was presented for 3750 ms, participants had to mark it as correct or incorrect by pressing the ‘d’ key (correct) or ‘k’ key (incorrect) on the keyboard. Following, a word was presented for 1250 ms to be stored in memory. The number sets of operation-word pairs increased along with the task from 2 to 6. After each set participants were asked to type all the words that they remembered from the set. To prevent recency effects participants were told that the only rule upon writing the set words was to avoid writing first the last word memorized, otherwise, the order of recall was not important. The logic behind this was to facilitate compliance with the task since it required participants to solve arithmetic operations and remembering words (see [59]), following the idea that a strict serial recall is not always required [64, 65]. The task was composed of 10 practice trials and 18 experimental trials (three trials per set size). A WM index was calculated by multiplying the number of successfully recalled words and the number of correctly solved equations [64]. The Spearman-Brown coefficient of reliability was 0.94.

*AX-CPT*. This task provides an index of the participants’ predominant mode of cognitive control (proactive vs. reactive). Specifically, we used the version described in Morales et al. [60], which has shown to be sensitive to individual differences. The task required participants to answer affirmatively to a specific pattern of letters, an ‘A’ letter (cue) followed by an ‘X’ letter (probe). To any other pattern of letters participants were required to answer ‘no’. Each letter was presented in the centre of the screen on a black background for 300 ms, with an inter-stimulus interval of 1000 ms. In every trial cue and probe were presented in red font. Additionally, between cue and probe, three letters were presented as distractors, in white font. Participants were instructed to respond ‘no’ to distractors. The task comprised 110 trials, 10 practice trials where feedback was provided, and 100 trials of the experimental block. The frequency of the target trials (AX) was 70%, while any other cue-probe combination (AY: ‘A’ cue–non ‘X’ probe; BX: non ‘A’ cue–‘X’ probe; or BY: neither ‘A’ cue nor ‘X’ probe) were presented in a 10% of the remaining cases. The tendency towards a control mode was assessed from the behavioural shift index [(BSI), computed as (AY-BX)/(AY+BX)] for errors [44, 50]. Higher scores in the BSI indicate greater dominance of proactive control, while lower scores indicate reactive control dominance. The index showed a split-half Spearman-Brown coefficient of 0.32.

## Results

Prior to performing analyses, we identified one participant with missing scores (in the BISS) and 6 participants who exhibited extremely poor performance (below 30% of accuracy in the cued task-switching or the working memory tasks) probably due to instructions misunderstanding. The scores of these participants in both tasks were excluded from the analysis [66]. The data of the study are available here: https://osf.io/cu2jt/?view_only=6c2779605e4a4309a1814e77058c12b4.

Descriptive statistics for the main variables of the study are presented in Table 1. General accuracy and RTs are reported along with the main indexes (and the trials that contribute to them) of each task. Namely, the switching cost index in the proactive possible block from the cued task-switching, the conflict cost from the conflict task, the working memory index derived from the working memory task (O-Span), and the Behavioural Shift Index (BSI) from the AX-CPT.

**Table 1:** Descriptive statistics of the scores from the personality questionnaires and experimental tasks. The first column refers to means (Ms) and standard deviations (SDs).

	Score	Minimum	Maximum
**Trait Scales**			
Grit-overall	3.41 (0.69)	1.75	4.87
Perseverance of effort	3.63 (0.69)	1.75	5
Consistency of interest	3.2 (0.89)	1	5
BISS	53.5 (19.54)	10	110
FFMQ	127.66 (18.35)	76	171
**Executive Tasks**			
*Cued Switching-Task*			
ACC	0.74 (0.14)	0.3	0.97
RT	676 (85)	383	886
No-switch trials	0.82 (0.18)	0.14	1
Switch trials	0.7 (0.22)	0.1	1
Switch Cost	0.13 (0.12)	-0.18	0.51
*Conflict Task*			
ACC	0.91 (0.11)	0.35	1
RT	724 (127)	445	1198
Congruent trials	689 (118)	435	1134
Incongruent trials	765 (146)	457	1281
Conflict Cost	75 (67)	-114	237
*Working Memory Task*			
Recalled words	0.84 (0.11)	0.45	1
ACC operations	0.74 (0.15)	0.35	1
Intrusions	0.04 (0.07)	0	0.75
Working Memory Index	0.63 (0.17)	0.2	0.98
*AX-CPT*			
ACC	0.88 (0.08)	0.65	0.99
RT	377 (81)	249	742
AY errors	0.27 (0.19)	0	0.8
BX errors	0.09 (0.15)	0	1
BY errors	0.02 (0.06)	0	0.3
BSI	0.13 (0.13)	-0.23	0.44

### Correlations analyses

We first performed Spearman correlations between the main variables of interest to identify potential predictors of grit. As can be seen in Table 2, the two personality traits highly correlated with grit and its facets (please, note that the correlation between BISS and Grit has been previously informed in [52], where BISS was used as a control variable to partial out the variance explained by this variable in the relationship between grit and the reported brain measures). Correlations between grit and the facets of the FFMQ are available in Supplementary Material 3 in S1 File. As for the variables obtained from the experimental tasks, higher grit (and consistency of interest) scores were linked to greater conflict cost and lower BSI. No relationships emerged between the Perseverance of effort facet of grit and the executive indexes. Although the relationship between the other personality variables and executive functioning is not the main focus of this work, they are reported here for transparency (see [51] for a discussion of the link between mindfulness and switching cost and BSI as calculated from RTs and accuracy).

**Table 2:** Spearman correlations between the main variables.

	Grit	Perseverance of Effort	Consistency of Interest	BISS	FFMQ	Switch Cost	Conflict Cost	Working Memory
Perseverance of Effort	0.83***							
Consistency of Interest	0.90***	0.52***						
BISS	-0.66***	-0.51***	-0.63***					
FFMQ	0.47***	0.48***	0.35***	-0.50***				
Switch Cost	-0.12	-0.10	-0.12	0.13	-0.19*			
Conflict Cost	0.22**	0.15	0.23**	-0.08	0.15	0.09		
Working Memory	-0.1	-0.16	-0.03	0.03	-0.02	-0.19*	-0.11	
BSI	-0.21*	-0.08	-0.26**	0.10	-0.14	-0.04	-0.19*	0.14

* p < .05

**p < .01

*** p < .001. Asterisks represent statistically significant correlations after controlling for multiple comparisons with the Banjamini-Hochberg method with false discovery rate at 0.1 (Benjamini & Hochberg, 1995 [67]).

### Factor and multiple regression analyses over the composite scores

Next, we run an exploratory factor analysis with the main variables of interest after transforming their values to Z scores. Thus, the total scores from the BISS and the FFMQ, the switching cost from the cued task-switching, and the corresponding indexes from the conflict and working memory tasks, and the AX-CPT were submitted to a factor analysis with a varimax rotation method, eigenvalue above 1.00 and maximum iterations for convergence set at 25 to reduce data and identify patterns in the measures.

Two factors resulted from the analysis that were also confirmed through visual inspection. Factor 1 accounted for 28% of the variance and was characterized by (low) mindfulness and (high) impulsiveness, whereas factor 2 accounted for 23.82% of the variance and included (high) working memory, (low) conflict cost, and (high) BSI (rotated solution). Factor loadings on the rotated solution are shown in Table 3.

**Table 3:** Factor loadings of the main indexes.

	*F1*	*F2*
BISS	**0.8**	0.1
FFMQ	**-0.83**	-0.13
Switch Cost (CS-T)	0.47	-0.52
Conflict Cost (CT)	-0.16	**-0.6**
Working Memory Index (O-Span)	-0.07	**0.66**
BSI (AX-CPT)	0.24	**0.58**

Since personality and executive indexes clustered in a two-factor structure and some of the executive indexes were not normally distributed (see Supplementary Material 4 in S1 File), we tested the relative weight of personality and executive components over grit by calculating a composite score from personality variables (impulsivity and mindfulness) and another from the executive indexes loading the most in the executive factor (working memory index, conflict cost and the BSI). Both composites showed good fit to normality (personality with *p* = 0.14 and executive with *p* = 0.18). The switching cost index was not included in any of the two composite scores because the index had similar weights in both factors (see Table 3). This composite-based approach allowed us to reduce complexity of the data and be able to run only one regression model with two predictors (executive and the personality composites). Composite scores were computed as the average of the z transformed scores of the indexes, with positive values representing better performance. Both composite scores were moderately and positively correlated (*r* = 0.36; *p* < 0.001).

Then, we run stepwise regression analyses over grit (and its facets) by using both composites as predictors. We included the executive composite first (despite the fact that the individual executive scores had a weaker association with grit than the personality scores) to better isolate its contribution after controlling for age, gender and education. Although the model with the executive composite reached statistical significance (and accounted for 6% of the variance in grit scores), the inclusion of the personality composite into the regression model accounted for 48% of the variance (see Table 4). In the model with the two factors, the executive composite negatively predicted grit (and consistency of interest) whereas the personality factor predicted grit (and its two facets) in a positive way. Partial correlation analyses revealed no association between grit and age, gender or education.

**Table 4:** Stepwise regression analysis of executive and personality composite scores over grit and its facets controlling for demographic variables.

	*R* ^*2*^	*ΔF*	*Β*	*SE*	*β*	*p*
***Grit***						
Model 1 (Executive)	0.06	8.21				0.005
Model 2 (both)	0.48	96.33				0.000
Executive Composite			-0.22	0.1	-0.14	0.033
Personality Composite			0.77	0.08	0.66	0.000
Excluded Variables				Partial Correlation		*P*
Age				0.04		0.9
Gender				0.06		1
Education				0.1		0.99
***Perseverance of Effort***						
Model 1 (Executive)	0.03	4.23				0.04
Model 2 (both)	0.35	56.63				0.000
Executive Composite			-0.14	0.12	-0.09	0.23
Personality Composite			0.65	0.09	0.57	0.000
Excluded Variables				Partial Correlation		*P*
Age				0.04		0.65
Gender				0.06		0.48
Studies				0.1		0.29
***Consistency of Interest***						
Model 1 (Executive)	0.06	8.25				0.005
Model 2 (both)	0.4	67.69				0.000
Executive Composite			-0.24	0.11	-0.16	0.032
Personality Composite			0.68	0.08	0.59	0.000
Excluded Variables				Partial Correlation		*P*
Age				0.00		0.9
Gender				0.16		1
Studies				0.1		0.99

## Discussion

The present study aimed to gain understanding of the relationship between executive functioning and grit also considering personality traits. More specifically, the main goal was to examine whether gritty individuals may be characterized in terms of executive functions. Thus, we selected two traits (impulsiveness and mindfulness) and four indexes of executive functioning (three from the framework proposed by Miyake et al. [28], and one from the dual mechanisms of cognitive control by Braver et al., [44]). We expected individuals scoring high on grit to exhibit enhanced executive functions, with improved ability for conflict resolution (better interference control), better working memory capacity, and enhanced proactive control and switching ability, since these processes should contribute to achieving long-term goals.

Our results replicate previous findings showing lower levels of impulsiveness and higher levels of mindfulness in people scoring high on grit [15, 18]. More relevant here, our results also show that executive functioning does not seem to be related to grit in the way it was predicted, since participants who scored high on grit did not exhibit better performance on the executive tasks than their low-scoring counterparts. Consistently, regression analyses showed that although the two composite scores derived from the factor analyses (trait and executive functioning) were predictive of grit, it was the personality composite (characterized by low impulsivity and high mindfulness) that explained more variance. Despite this, the executive composite (characterized by low conflict cost, high working memory, and high BSI) was negatively and reliably related to grit. Hence, our results suggest that the relation between executive functions and grit might be more complex than predicted and go in line with Duckworth’s suggestion that high grit is not necessarily characterized by better executive functioning [3, 23, 24]. Additionally, our results agree with findings from previous studies showing regulation-related personality traits (low impulsiveness and high mindfulness) to have a strong link with the development and expression of grit [15, 18].

Although grit seems not to be related to enhanced cognitive functioning, the present results might be indicating certain patterns in the use of cognitive control by gritty individuals. The regression analyses showed that the executive indexes (in the form of a composite score) accounted for a small percentage of grit variance. More specifically, correlation analyses showed that the conflict cost (an inverse index of the ability to control interference) and the BSI (an index thought to reflect the relative weight of proactive/reactive cognitive control) were significantly, even though weakly, associated with grit. The BSI was negatively related to grit and its facet of consistency of interest, which indicates less proactive control in gritty participants and is contrary to our expectations. In a similar vein, the conflict cost positively correlated with grit and the consistency of interest facet, which suggests that interference control might be less efficient in gritty participants. In this regard, although grit has shown to be linked to self-control related traces in the brain [34–37], its relationship with executive-control indexes from experimental tasks has been scarcely investigated [25]. One possible explanation of this discrepancy is the lack of correspondence between the self-reported measures of self-control and the executive-control indexes derived from experimental tasks [i.e., see, 68, 69], as well as between brain activity and performance. Thus, for example, previous results have showed grit to be linked to electrophysiological activity thought to reflect better effortful control in a learning task, but not to better learning performance [52].

Our finding of lower BSI with higher grit aligns with the results by Kalia et al. [25] showing a negative association between grit and the alerting effect, whereby high grit participants benefited less from the presence of a contextual alerting cue. We posit that both findings may be indicating that gritty individuals, relative to those scoring low on grit, keep attentive to all available information, what makes them to rely less on previous/current contextual information driving to a fast answer (lower alerting effect and lower BSI), and slowing their response when the information is contradictory (higher conflict cost). This could help them to prevent automatic responses and then to achieve long-term superordinate goals, even though it would not be particularly beneficial for immediate performance. It must be highlighted, however, that the relevance of these cognitive variables in predicting grit is negligible when compared to the personality traits considered in the present study, which is in line with previous findings in the literature [25]. Thus, overall, our results support the notion that gritty people do not exhibit better executive functioning [3, 23, 24].

As for the positive association between mindfulness and grit, our results replicate finding by others with different ethnical groups from the United States, New Zealand, Thailand and China [17, 19, 70, 71]. Interestingly, at the facet level, our Spanish sample was similar to the New Zealand’s sample in the study by Raphiphatthana et al. [19], so that the two facets of grit were positively related to the acting with awareness facet and the perseverance of effort facet of grit was specifically related to the non-judging facet of mindfulness (for a deep discussion on the role of grit and mindfulness in western and eastern countries see [19]). Interestingly, the present-oriented attention of high mindful individuals fits well with our finding on the less proactive mode of control of gritty participants. In fact, this has also been reported to be characteristic of high mindfulness trait [51, 72]. Hence, one might think that impulsivity and mindfulness are mediating the (weak) effects of executive functioning over grit. However, it should be noted that the relationship between the two composite scores (executive and personality) is positive (positive traits are linked to better executive functioning), whereas the prediction of grit by executive functioning is negative. Thus, it would seem that the link between a certain profile of executive functioning and grit is independent of the other two personality traits considered here.

In summary, altogether our results go along with the idea that enhanced cognitive ability is not a characteristic of gritty people. Instead, and relative to their low grit counterparts, they exhibit a different pattern of performance that might be reflecting a cautious profile of control, characterized by paying attention to all available information, less reliance on previous contextual cues but sensitive to conflicting information of the current context. This could help them to be more aware of the presence of conflicting goals in their everyday life. This seems a plausible cognitive profile that fits well with the main finding that less impulsivity and more mindfulness traits account for a large amount of grit variance.

The present study is not without limitations. Its exploratory and cross-sectional nature makes it necessary to be cautious regarding the observed associations. Future research with longitudinal and/or intervention-based approaches could help in further specifying the nature of the link between trait-executive functioning profiles and grit. Importantly, future works should include measures of fluid intelligence (as it could influence working memory [73]) and use more complete measures of grit (to overcome the reliability issues of the short grit scale [74]). Despite this, the present study contributes to our understanding of grit and provides strong evidence for the idea that it is not necessarily linked to enhanced executive-control capabilities [3, 23, 24]. Rather, high grit seems to entail a different (more cautious) cognitive control mode.

## Supporting information

S1 File (DOCX)

## References

[1] Duckworth A, Gross JJ. Self-Control and Grit: Related but Separable Determinants of Success. Curr Dir Psychol Sci. 2014;23(5):319–25. doi: 10.1177/0963721414541462 26855479 PMC4737958

[2] Seligman MEP, Duckworth A. Research Article: Self-Discipline Outdoes IQ in Predicting Academic Performance of Adolescents. Psychol Sci. 2005;16(12):939–44. doi: 10.1111/j.1467-9280.2005.01641.x 16313657

[3] Duckworth AL, Peterson C, Matthews MD, Kelly DR. Grit: Perseverance and Passion for Long-Term Goals. J Pers Soc Psychol. 2007;92(6):1087–101. doi: 10.1037/0022-3514.92.6.1087 17547490

[4] Jiang W, Jiang J, Du X, Gu D, Sun Y, Zhang Y. Striving and happiness: Between- and within-person-level associations among grit, needs satisfaction and subjective well-being. J Posit Psychol [Internet]. 2020;15(4):543–55. Available from: 10.1080/17439760.2019.1639796

[5] Datu JAD, King RB, Valdez JPM, Eala MSM. Grit is Associated with Lower Depression via Meaning in Life among Filipino High School Students. Youth Soc. 2019;51(6):865–76.

[6] Musumari PM, Tangmunkongvorakul A, Srithanaviboonchai K, Techasrivichien T, Suguimoto SP, Ono-Kihara M, et al. Grit is associated with lower level of depression and anxiety among university students in Chiang Mai, Thailand: A cross-sectional study. PLoS One. 2018;13(12):1–16. 10.1371/journal.pone.0209121 PMC6294431 30550597

[7] Kleiman EM, Adams LM, Kashdan TB, Riskind JH. Gratitude and grit indirectly reduce risk of suicidal ideations by enhancing meaning in life: Evidence for a mediated moderation model. J Res Pers. 2013;47(5):539–46.

[8] White EJ, Kraines MA, Tucker RP, Wingate LRR, Wells TT, Grant DMM. Rumination’s effect on suicide ideation through grit and gratitude: A path analysis study. Psychiatry Res. 2017;251(January):97–102. doi: 10.1016/j.psychres.2017.01.086 28199915

[9] Eskreis-Winkler L, Shulman EP, Beal SA, Duckworth AL. The grit effect: Predicting retention in the military, the workplace, school and marriage. Front Psychol. 2014;5(FEB):1–12. doi: 10.3389/fpsyg.2014.00036 24550863 PMC3910317

[10] Mueller BA, Wolfe MT, Syed I. Passion and grit: An exploration of the pathways leading to venture success. J Bus Ventur. 2017;32(3):260–79. Available from: 10.1016/j.jbusvent.2017.02.001

[11] Clark RS, Clark VL. Grit within the Context of Career Success: a Mixed Methods Study. Int J Appl Posit Psychol. 2019;4(3):91–111.

[12] Duckworth AL, Quinn PD. Development and validation of the short Grit Scale (Grit-S). J Pers Assess. 2009;91(2):166–74. doi: 10.1080/00223890802634290 19205937

[13] Muenks K, Wigfield A, Yang JS, O’Neal CR. How true is grit? Assessing its relations to high school and college students’ personality characteristics, self-regulation, engagement, and achievement. J Educ Psychol. 2017;109(5):599–620.

[14] Werner KM, Milyavskaya M, Klimo R, Levine SL. Examining the unique and combined effects of grit, trait self-control, and conscientiousness in predicting motivation for academic goals: A commonality analysis. J Res Pers [Internet]. 2019;81:168–75. Available from: 10.1016/j.jrp.2019.06.003

[15] Griffin ML, McDermott KA, McHugh RK, Fitzmaurice GM, Weiss RD. Grit in patients with substance use disorders. Am J Addict. 2016;25(8):652–8. doi: 10.1111/ajad.12460 27759947 PMC5484735

[16] Moshier SJ, Szuhany KL, Hearon BA, Smits JAJ, Otto MW. Anxiety Sensitivity Uniquely Predicts Exercise Behaviors in Young Adults Seeking to Increase Physical Activity. 2016; doi: 10.1177/0145445515603704 26342011

[17] Li J, Lin L, Zhao Y, Chen J, Wang S. Grittier Chinese adolescents are happier: The mediating role of mindfulness. Pers Individ Dif [Internet]. 2018;131(April):232–7. Available from: 10.1016/j.paid.2018.05.007

[18] Raphiphatthana B, Jose P, Salmon K. Does Dispositional Mindfulness Predict the Development of Grit? https://doi.org/101027/1614-0001/a000252 [Internet]. 2018 Apr 4 [cited 2021 Sep 4];39(2):76–87. Available from: https://econtent.hogrefe.com/doi/abs/10.1027/1614-0001/a000252

[19] Raphiphatthana B, Jose PE, Chobthamkit P. The Association Between Mindfulness and Grit: an East vs. West Cross-cultural Comparison. Mindfulness (N Y). 2019;10(1):146–58.

[20] Raphiphatthana B, Jose PE. The Relationship Between Dispositional Mindfulness and Grit Moderated by Meditation Experience and Culture. Mindfulness (N Y). 2020;11(3):587–98.

[21] Kabat-Zinn J. Mindfulness-based stress reduction (MBSR). Constr Hum Sci [Internet]. 2003;8((2)):73. Available from: 10.1016/j.jaci.2012.05.050

[22] Baer RA, Smith GT, Hopkins J, Krietemeyer J, Toney L. Using self-report assessment methods to explore facets of mindfulness. Assessment. 2006;13(1):27–45. doi: 10.1177/1073191105283504 16443717

[23] Perkins-Gough D. The significance of grit: A conversation with Angela Lee Duckworth. Educ Leadersh. 2013;71(1):14–20.

[24] Credé M, Tynan MC, Harms PD. Much ado about grit: A meta-analytic synthesis of the grit literature. J Pers Soc Psychol. 2017;113(3):492–511. doi: 10.1037/pspp0000102 27845531

[25] Kalia V, Thomas R, Osowski K, Drew A. Staying alert? Neural correlates of the association between Grit and Attention Networks. Front Psychol. 2018;9(AUG):1–14. doi: 10.3389/fpsyg.2018.01377 30123173 PMC6085581

[26] Fan J, McCandliss BD, Sommer T, Raz A, Posner MI. Testing the efficiency and independence of attentional networks. J Cogn Neurosci. 2002;14(3):340–7. doi: 10.1162/089892902317361886 11970796

[27] Kalia V, Fuesting M, Cody M. Perseverance in solving Sudoku: role of grit and cognitive flexibility in problem solving. J Cogn Psychol [Internet]. 2019;31(3):370–8. Available from: 10.1080/20445911.2019.1604527

[28] Miyake A, Friedman NP, Emerson MJ, Witzki AH, Howerter A, Wager TD. The Unity and Diversity of Executive Functions and Their Contributions to Complex “Frontal Lobe” Tasks: A Latent Variable Analysis. Cogn Psychol. 2000;41(1):49–100. doi: 10.1006/cogp.1999.0734 10945922

[29] Hofmann W, Schmeichel BJ, Baddeley AD. Executive functions and self-regulation. Trends Cogn Sci [Internet]. 2012;16(3):174–80. Available from: doi: 10.1016/j.tics.2012.01.006 22336729

[30] Hinson JM, Jameson TL, Whitney P. Impulsive Decision Making and Working Memory. J Exp Psychol Learn Mem Cogn [Internet]. 2003 Mar [cited 2021 Sep 4];29(2):298–306. Available from: /record/2003-02055-011 doi: 10.1037/0278-7393.29.2.298 12696817

[31] Anicha CL, Ode S, Moeller SK, Robinson MD. Toward a Cognitive View of Trait Mindfulness: Distinct Cognitive Skills Predict Its Observing and Nonreactivity Facets. J Pers. 2012;80(2):255–85. doi: 10.1111/j.1467-6494.2011.00722.x 21299556

[32] Verhaeghen P. Mindfulness as Attention Training: Meta-Analyses on the Links Between Attention Performance and Mindfulness Interventions, Long-Term Meditation Practice, and Trait Mindfulness. Mindfulness (N Y). 2021;12(3):564–81.

[33] Yuan P, Raz N. Prefrontal cortex and executive functions in healthy adults: A meta-analysis of structural neuroimaging studies. Neurosci Biobehav Rev [Internet]. 2014;42:180–92. Available from: doi: 10.1016/j.neubiorev.2014.02.005 24568942 PMC4011981

[34] Myers CA, Wang C, Black JM, Bugescu N, Hoeft F. The matter of motivation: Striatal resting-state connectivity is dissociable between grit and growth mindset. Soc Cogn Affect Neurosci. 2016;11(10):1521–7. doi: 10.1093/scan/nsw065 27217105 PMC5040906

[35] Nemmi F, Nymberg C, Helander E, Klingberg T. Grit Is Associated with Structure of Nucleus Accumbens and Gains in Cognitive Training. 2016;(1):1688–99. 10.1162/jocn_a_01031 27626223

[36] Wang S, Zhou M, Chen T, Yang X, Chen G, Wang M, et al. Grit and the brain: Spontaneous activity of the dorsomedial prefrontal cortex mediates the relationship between the trait grit and academic performance. Soc Cogn Affect Neurosci. 2017;12(3):452–60. doi: 10.1093/scan/nsw145 27672175 PMC5390743

[37] Wang S, Dai J, Li J, Wang X, Chen T, Yang X, et al. Neuroanatomical correlates of grit: Growth mindset mediates the association between gray matter structure and trait grit in late adolescence. Hum Brain Mapp. 2018;39(4):1688–99. doi: 10.1002/hbm.23944 29331059 PMC6866491

[38] Miyake A, Friedman NP. The nature and organization of individual differences in executive functions: Four general conclusions. Curr Dir Psychol Sci. 2012;21(1):8–14. doi: 10.1177/0963721411429458 22773897 PMC3388901

[39] Chevalier N., Martis S. B., Curran T., & Munakata Y. Metacognitive processes in executive control development: The case of reactive and proactive control. J Cogn Neurosci [Internet]. 2015;27(6):1125–36. Available from: https://www.apa.org/ptsd-guideline/ptsd.pdf%0Ahttps://www.apa.org/about/offices/directorates/guidelines/ptsd.pdf doi: 10.1162/jocn_a_00782 25603026 PMC4510990

[40] Monsell S. Task switching. 2003;7(3):134–40. doi: 10.1016/s1364-6613(03)00028-7 12639695

[41] Roelofs A, Van Turennout M, Coles MGH. Anterior cingulate cortex activity can be independent of response conflict in Stroop-like tasks. Proc Natl Acad Sci U S A. 2006;103(37):13884–9. doi: 10.1073/pnas.0606265103 16954195 PMC1564252

[42] Turner ML, Engle RW. Is working memory capacity task dependent? J Mem Lang. 1989;28(2):127–54.

[43] Friedman NP, Miyake A, Young SE, DeFries JC, Corley RP, Hewitt JK. Individual Differences in Executive Functions Are Almost Entirely Genetic in Origin. J Exp Psychol Gen. 2008;137(2):201–25. doi: 10.1037/0096-3445.137.2.201 18473654 PMC2762790

[44] Braver TS, Paxton JL, Locke HS, Barch DM. Flexible neural mechanisms of cognitive control within human prefrontal cortex. Proc Natl Acad Sci U S A. 2009;106(18):7351–6. doi: 10.1073/pnas.0808187106 19380750 PMC2678630

[45] Braver TS. The variable nature of cognitive control: A dual mechanisms framework. Trends Cogn Sci [Internet]. 2012;16(2):106–13. Available from: doi: 10.1016/j.tics.2011.12.010 PMC3289517 22245618

[46] Jimura K, Locke HS, Braver TS. Prefrontal cortex mediation of cognitive enhancement in rewarding motivational contexts. Proc Natl Acad Sci U S A. 2010;107(19):8871–6. doi: 10.1073/pnas.1002007107 20421489 PMC2889311

[47] Burgess GC, Braver TS. Neural mechanisms of interference control in working memory: Effects of interference expectancy and fluid intelligence. PLoS One. 2010;5(9):1–11. doi: 10.1371/journal.pone.0012861 20877464 PMC2942897

[48] Mäki-Marttunen V, Hagen T, Espeseth T. Task context load induces reactive cognitive control: An fMRI study on cortical and brain stem activity. Cogn Affect Behav Neurosci. 2019;19(4):1094. doi: 10.3758/s13415-019-00701-7 30659515 PMC6711881

[49] Munakata Y, Snyder HR, Chatham CH. Developing Cognitive Control: Three Key Transitions. Curr Dir Psychol Sci. 2012;21(2):71–7. doi: 10.1177/0963721412436807 22711982 PMC3375849

[50] Gómez-Ariza CJ, Martín MC, Morales J. Tempering proactive cognitive control by transcranial direct current stimulation of the right (but not the left) lateral prefrontal cortex. Front Neurosci. 2017;11(MAY):1–12. doi: 10.3389/fnins.2017.00282 28588441 PMC5440549

[51] Aguerre N V., Bajo MT, Gómez-Ariza CJ. Dual mechanisms of cognitive control in mindful individuals. Psychol Res [Internet]. 2020;(0123456789). Available from: doi: 10.1007/s00426-020-01377-2 32638070

[52] Aguerre N V., Gómez-Ariza CJ., Ibáñez-Molina A J., Bajo MT. Electrophysiological Prints of Grit. Frontiers in psychology 2021, 12. doi: 10.3389/fpsyg.2021.730172 34721192 PMC8551368

[53] Erdfelder E, Faul F, Buchner A. GPOWER: A general power analysis program. Behav Res Methods, Instruments, Comput. 1996;28(1):1–11.

[54] Association WM. World Medical Association Declaration of Helsinki: Ethical Principles for Medical Research Involving Human Subjects. JAMA [Internet]. 2013 Nov 27 [cited 2021 Sep 4];310(20):2191–4. Available from: https://jamanetwork.com/journals/jama/fullarticle/1760318 doi: 10.1001/jama.2013.281053 24141714

[55] Oquendo M. A., Baca-García E., Graver R., Morales M., Montalvan V., & Mann J. Spanish adaptation of the Barratt impulsiveness scale (BIS-11). Eur J Psychiatry. 2001;15(3):147–55.

[56] Cebolla A, García-Palacios A, Soler J, Guillen V, Baños R, Botella C. Psychometric properties of the Spanish validation of the Five Facets of Mindfulness Questionnaire (FFMQ). Eur J Psychiatry. 2012;26(2):118–26.

[57] Soler J., Tejedor R., Feliu-Soler A., Pascual J. C., Cebolla A., & Soriano J. Propiedades psicométricas de la versión española de la escala Mindful Attention Awareness Scale (MAAS). Actas Esp Psiquiatr. 2012;40(Barcelona 08025):18–25. 22344492

[58] Stroop. Of, Studies In, Interference Reactions, Verbal. Journal of Experimental Psychology. 1935;XVIII(6).

[59] Maraver MJ, Bajo MT, Gomez-Ariza CJ. Training on working memory and inhibitory control in young adults. Front Hum Neurosci. 2016;10(NOV2016):1–18. 27917117 10.3389/fnhum.2016.00588 PMC5114277

[60] Morales J, Gómez-Ariza CJ, Bajo MT. Dual mechanisms of cognitive control in bilinguals and monolinguals. J Cogn Psychol. 2013;25(5):531–46.

[61] Verbruggen F, Logan GD. Response inhibition in the stop-signal paradigm. Trends Cogn Sci. 2008;12(11):418–24. doi: 10.1016/j.tics.2008.07.005 18799345 PMC2709177

[62] Anderson MC, Bjork RA, Bjork EL. Remembering Can Cause Forgetting: Retrieval Dynamics in Long-Term Memory. J Exp Psychol Learn Mem Cogn. 1994;20(5):1063–87. doi: 10.1037//0278-7393.20.5.1063 7931095

[63] Schneider W., Eschman A., & Zuccolotto A. E-Prime: User’s Guide. Reference Guide. Getting Started Guide. Psychol Softw Tools, Inc.

[64] Conway ARA, Kane MJ, Bunting MF, Hambrick DZ, Wilhelm O, Engle RW. Working memory span tasks: A methodological review and user’s guide. Psychon Bull Rev 2005 125 [Internet]. 2005 [cited 2021 Sep 4];12(5):769–86. Available from: https://link.springer.com/article/10.3758/BF03196772 16523997 10.3758/bf03196772

[65] Friedman NP, Miyake A. The reading span test and its predictive power for reading comprehension ability. J Mem Lang. 2004;51(1):136–58.

[66] Gonthier C, Braver TS, Bugg JM. Dissociating proactive and reactive control in the Stroop task. Mem Cognit [Internet]. 2016;(February):778–88. Available from: http://link.springer.com/10.3758/s13421-016-0591-1 26861210 10.3758/s13421-016-0591-1 PMC4942492

[67] Benjamini Yoav, and Hochberg Yosef. "Controlling the false discovery rate: a practical and powerful approach to multiple testing." Journal of the Royal statistical society: series B (Methodological) 57.1 (1995): 289–300.

[68] Nȩcka E, Gruszka A, Orzechowski J, Nowak M, Wójcik N. The (In)significance of executive functions for the trait of self-control: A psychometric study. Front Psychol. 2018;9(JUL):1–12. 30038592 10.3389/fpsyg.2018.01139 PMC6046447

[69] Saunders B, Milyavskaya M, Etz A, Randles D, Inzlicht M. Reported Self-control is not Meaningfully Associated with Inhibition-related Executive Function: A Bayesian analysis. Collabra Psychol. 2018;4(1):1–16.30637365

[70] Brennan BM, Ketcham CJ, Patel K, Hall EE, Viana BF, Ribeiro B, et al. May 31 9: 00 AM—10: 30 AM Academic Confidence and Grit Predict Mindfulness in Collegiate Student-Athletes May 31 9: 00 AM—10: 30 AM Psychophysiological And Pacing Strategy Responses To A Sprint Exercise Performed With Different Exercise Expectatio. 2018;2018.

[71] Vela JC, Smith WD, Whittenberg JF, Guardiola R, Savage M. Positive Psychology Factors as Predictors of Latina/o College Students’ Psychological Grit. J Multicult Couns Devel. 2018;46(1):2–19.

[72] Chang JH, Kuo CY, Huang CL, Lin YC. The Flexible Effect of Mindfulness on Cognitive Control. Mindfulness (N Y). 2018;9(3):792–800.

[73] Luciano M, Wright MJ, Smith GA, Geffen GM, Geffen LB, Martin NG. Genetic covariance among measures of information processing speed, working memory, and IQ. Behav Genet. 2001;31(6):581–92. doi: 10.1023/a:1013397428612 11838535

[74] Kuruveettissery S, Gupta S, Rajan SK. Development and psychometric validation of the three dimensional grit scale. Curr Psychol. 2021.

