# Supplementary material for: The relative role of executive control and personality traits in grit
Source: PLoS One. 2022 Jun 22;17(6):e0269448. doi: 10.1371/journal.pone.0269448 (PMC9216537; doi:10.1371/journal.pone.0269448)
Supplement: S1 File — (DOCX) [file pone.0269448.s001.docx]

# Supplementary Material 1

Table 1 Factor Analysis over Grit Scale items. *Kaiser-Meyer-Olkin Measure* of 0.84 and *p* = 0.000.

|  | *F1* | *F2* |
| --- | --- | --- |
| Item 1 (CI) | 0.82 | 0.06 |
| Item 2 (PE) | 0.08 | 0.65 |
| Item 3 (CI) | 0.74 | -0.15 |
| Item 4 (PE) | -0.44 | 0.6 |
| Item 5 (CI) | 0.78 | -0.1 |
| Item 6 (CI) | 0.77 | -0.18 |
| Item 7 (PE) | -0.69 | 0.39 |
| Item 8 (PE) | -0.2 | 0.72 |

# Supplementary Material 2

Table 1 Matrix correlation of grit with all measures of the Cued Switching-Task.

|  | Grit | Perseverance of Effort | Consistency of Interest |
| --- | --- | --- | --- |
| Cued Switching-Task |  |  |  |
| ACC | -0.06 | -0.01 | -0.07 |
| RT | 0.17 | 0.18 | 0.16 |
| ACC P. Possible | 0.02 | 0.04 | -0.00 |
| RT P. Possible | 0.13 | 0.12 | 0.14 |
| SC P. Possible based on ACC | -0.12 | -0.1 | -0.12 |
| SC P. Possible based on RT | -0.00 | -0.00 | 0.03 |
| ACC P. Encouraged | -0.14 | -0.15 | -0.09 |
| RT P. Encouraged | 0.07 | 0.13 | 0.04 |
| SC P. Encouraged based on ACC | 0.16 | 0.23 | 0.07 |
| SC P. Encouraged based on RT | 0.02 | 0.03 | 0.01 |
| ACC P. Impossible | 0.03 | 0.15 | -0.05 |
| RT P. Impossible | 0.13 | 0.15 | 0.14 |
| SC P. Impossible based on ACC | 0.02 | 0.05 | 0.01 |
| SC P. Impossible based on RT | 0.14 | 0.08 | 0.14 |

* p < .05 **p < .01, *** p < .001. Asterisks represent statistically significant correlations after controlling for multiple comparisons with the Banjamini-Hochberg method with false discovery rate at .1 (Benjamini & Hochberg, 1995 [67]).

# Supplementary Material 3

Table 1 Correlation matrix between grit and FFMQ and their facets.

|  | Grit Total | Perseverance of Effort | Consistency of Interest |
| --- | --- | --- | --- |
| FFMQ | 0.47*** | 0.46*** | 0.38*** |
| Observing | 0.08 | 0.19* | -0.01 |
| Describing | 0.27** | 0.28** | 0.22* |
| Acting Awareness | 0.58*** | 0.42*** | 0.56*** |
| Non-Judging | 0.18* | 0.15 | 0.14 |
| Non-Reactivity | 0.21* | 0.31*** | 0.08 |

* p < .05 **p < .01, *** p < .001. Asterisks represent statistically significant correlations after controlling for multiple comparisons with the Banjamini-Hochberg method with false discovery rate at .1 (Benjamini & Hochberg, 1995 [67]).

# Supplementary Material 4

Table 1 Normality analyses over the variables of interest.

|  | Shapiro-Wilk | *p* |
| --- | --- | --- |
| Grit | 0.98 | 0.052 |
| Perseverance of effort | 0.97 | 0.006 |
| Consistency of interest | 0.97 | 0.02 |
| FFMQ | 0.99 | 0.24 |
| BISS | 0.98 | 0.06 |
| WM Index (O-Span) | 0.98 | 0.02 |
| Conflict Cost | 0.99 | 0.42 |
| BSI (AX-CPT) | 0.98 | 0.06 |
